# Enhanced Photocatalytic Hydrogen Production of the Polyoxoniobate Modified with RGO and PPy

**DOI:** 10.3390/nano10122449

**Published:** 2020-12-07

**Authors:** Shiliang Heng, Lei Li, Weiwei Li, Haiyan Li, Jingyu Pang, Mengzhen Zhang, Yan Bai, Dongbin Dang

**Affiliations:** 1 Henan Key Laboratory of Polyoxometalate Chemistry, College of Chemistry and Chemical Engineering, Henan University, Kaifeng 475004, China; 104753170767@vip.henu.edu.cn (S.H.); leili1202@126.com (L.L.); 104753160776@vip.henu.edu.cn (W.L.); lihaiyan@henu.edu.cn (H.L.); pjy@henu.edu.cn (J.P.); 104754181031@vip.henu.edu.cn (M.Z.); 2 College of Chemistry and Chemical Engineering, Anyang Normal University, Anyang 455002, China

**Keywords:** polyoxoniobate, Nb_6_/PPy-RGO composites, electronic bridge, H_2_ production

## Abstract

The development of high-efficiency, recyclable, and inexpensive photocatalysts for water splitting for hydrogen production is of great significance to the application of solar energy. Herein, a series of graphene-decorated polyoxoniobate photocatalysts Nb_6_/PPy-RGO (Nb_6_ = K_7_HNb_6_O_19_, RGO = reduced graphene oxide, PPy = polypyrrole), with the bridging effect of polypyrrole were prepared through a simple one-step solvothermal method, which is the first example of polyoxoniobate-graphene-based nanocomposites. The as-fabricated photocatalyst showed a photocatalytic H_2_ evolution activity without any co-catalyst. The rate of 1038 µmol g^−1^ in 5 h under optimal condition is almost 43 times higher than that of pure K_7_HNb_6_O_19_·13H_2_O. The influencing factors for photocatalysts in photocatalytic hydrogen production under simulated sunlight were studied in detail and the feasible mechanism is presented in this paper. These results demonstrate that Nb_6_O_19_ acts as the main catalyst and electron donor, RGO provides active sites, and PPy acted as an electronic bridge to extend the lifetime of photo-generated carriers, which are crucial factors for photocatalytic H_2_ production.

## 1. Introduction

With the widespread energy consumption and environmental problems, it is necessary and urgent to search for sources of green and clean energy to replace traditional fossil fuels [1,2,3,4]. Since the first discovery of photoelectrochemical water splitting into H_2_ via the semiconductor TiO_2_ by Fujishima and Honda in 1972, photocatalytic water splitting to hydrogen production has long been viewed as a promising and attractive strategy [5]. In recent years, many semiconductor materials have been reported for photocatalytic hydrogen evolution, in which the band gap, the position of the conduction band, the separation of electrons and holes and the lifetime of carriers are the key factors affecting photocatalysis [6,7]. Nonetheless, the exploitation of highly effective semiconductor materials for photocatalytic hydrogen evolution is still in the infant stage [8,9,10].

Polyoxometalates (POMs) have been applied in photocatalytic H_2_ or O_2_ evolution and the photodegradation of organic dyes [11,12,13,14,15,16], due to their controllable and stable structures, superior redox and semiconductor properties [17,18,19,20,21,22]. However, the lower H_2_ production rates and the introduction of the scarce noble metal have largely limited the scale of their potential applications. It is a feasible strategy to design and prepare photocatalyst composites based on POMs, such as [PW_12_O_40_]^3−^, [PMo_12_O_40_]^3−^, [SiW_12_O_40_]^4−^ and [PMo_10_V_2_O_40_]^5−^ [23,24,25,26,27,28,29,30]. In 2018, Zhang reported an interesting investigation on the preparation of a novel Ag_3_PO_4_/POM/graphene oxide (GO) heterostructure photocatalyst based on molybdophosphoric acid, which has excellent photocatalytic activity for photodegraded rhodamine B and water splitting for H_2_ evolution, because of its increased surface area, electronegativity and structural stability [28]. This is a pioneering work in the study of photocatalyst composites from well conductive graphene and POMs for H_2_ production.

Graphene is an ideal support material and as a solid electron mediator in photocatalysis, owing to the special 2D sp^2^-hybridied honeycomb structure [2,31], high charge carrier mobility (ca. 10,000 cm^2^ V^−1^ s^−1^) and large theoretical surface area (2600 m^2^ g^−1^) [32,33,34]. Therefore, the combination of graphene with semiconductors can not only inhibit the recombination of the photogenerated electron-hole pairs, but also adjust the band gap of photocatalysts and provide more photoactive sites [35,36,37]. More importantly, functionalization of N-doped graphene is a good way to improve the photocatalytic behavior. In particular, pyrrole-N-doped graphene is used as an oxygen-reduction active site to accelerate the interface-catalytic process [38]. Therefore, it is a promising work to introduce pyrrole into POM and graphene-based composites for photocatalytic decomposition of water into H_2_O_2_ or hydrogen and oxygen.

As an important kind of POMs, polyoxoniobates have been widely used in photocatalytic H_2_ evolution due to higher negative charges and stronger basicity of [Nb_6_O_19_]^8−^, which can easily accept electrons and keep an intact structure. Recently, several studies on photocatalytic H_2_ evolution by polyoxoniobates have been reported, in which precious metals are essential components as co-catalysts [39,40,41,42,43]. Taking these studies into account, we hope to design a new type of POM-based composite photocatalysts with good photocatalytic H_2_ production performance without any co-catalyst. Therefore, graphene was introduced into [Nb_6_O_19_]^8−^ for the first time to produce composite photocatalysts, where it could act as the active sites to produce H_2_, avoiding the use of the scarce noble metal, while using economical and non-toxic pyrrole as a bridging material to prolong the lifetime of photo-generated carriers [44,45], decreasing the recombination of electron-hole pairs. Herein, Nb_6_/PPy-RGO (Nb_6_ = K_7_HNb_6_O_19_, RGO = reduced graphene oxide, PPy = polypyrrole), composites with the appearance of peony flowers were prepared using a simple one-pot solvothermal process. By adjusting the molar rate of Nb_6_, the volume of pyrrole and solvothermal temperature, a series of Nb_6_/PPy-RGO nanocomposites were obtained. Among the achieved Nb_6_/PPy-RGO composites, Nb_6_/PPy-RGO-0.25 shows an outstanding photocatalytic activity with H_2_ production rate of 1038 µmol g^−1^ in 5 h under the simulated sunlight without co-catalyst. Moreover, as-obtained Nb_6_/PPy-RGO composites demonstrate high stability and superb recyclability, which can be attributed to the collaborations among Nb_6_, RGO and PPy.

## 2. Materials and Methods 

### 2.1. Materials

All the reagents and solvents used in this experiment were reagent grade and were used without further treatment. Graphite powder, sulfuric acid (H_2_SO_4_, 98%), sodium sulfate (Na_2_SO_4_, 99%), sodium nitrate (NaNO_3_, 99%), potassium permanganate (KMnO_4_, 99.5%) and pyrrole (C_4_H_5_N, 98%) were purchased from Aladdin Biochemical Technology Co. (Shanghai, China). The K_7_HNb_6_O_19_·13H_2_O was synthesized according to the method in the literature [46], and its purity was determined by infrared spectra (IR). Graphene oxide (GO) was manufactured by a modified Hummers’ method and the purity of GO was confirmed by X-ray diffraction (XRD) [47,48].

### 2.2. Synthesis of Nb_6_/PPy-RGO

**Scheme 1:**
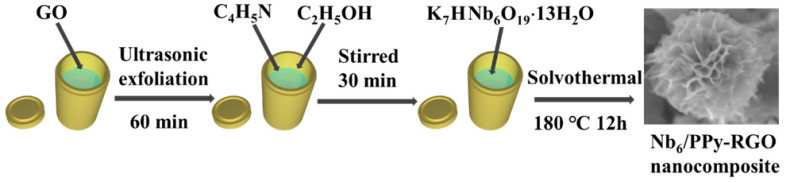
Illustration of the synthesis process of Nb_6_/PPy-RGO composites (K_7_HNb_6_O_19_·13H_2_O = Nb_6_, RGO = reduced graphene oxide, PPy = polypyrrole).

As shown in Scheme 1, 4 mg graphene oxide (GO) was added to 2 mL distilled water in a 25 mL Teflon lining and ultrasonic for 1 h. Then, a solution containing 110 μL pyrrole (denoted as Py) and 1 mL ethanol was added to the above solution., After stirring for 30 min, Nb_6_ (345 mg, 0.25 mmol) was added to the mixed solution and heated at 180 °C for 12 h. After cooling to room temperature, the achieved product, Nb_6_/PPy-RGO-0.25, was respectively washed with deionized water four times and ethanol two times to remove the excess reactants, and finally dried at 60 °C under vacuum for 12 h. According to the same procedure, except for the amount of Nb_6_ (173 mg, 0.125 mmol) and (523 mg, 0.375 mmol), the samples Nb_6_/PPy-RGO-0.125 and Nb_6_/PPy-RGO-0.375 were respectively obtained. Similarly, to gain further insight into the effect of the amount of Nb_6_ and reaction temperature on the photocatalytic activity of the catalysts, controlling the amount of Nb_6_ (0.25 mmol), GO and temperature, we achieved Nb_6_/PPy-RGO-90 and Nb_6_/PPy-RGO-130 by adjusting the amount of pyrrole (90 and 130 μL). Simultaneously, controlling the amount of Nb_6_ (0.25 mmol), GO and pyrrole (110 μL), the samples Nb_6_/PPy-RGO-160 °C and Nb_6_/PPy-RGO-190 °C were fabricated. As a comparison, the PPy-RGO was also obtained without the addition of Nb_6_.

### 2.3. Characterization

The morphology and microstructure of the synthetic samples were characterized using a field emission scanning electron microscopy (FESEM, JSM-7610F, Electronics Co., LTD., Tokyo, Japan) at 10 kV and a transmission electron microscopy (TEM, JEM-2100F, Electronics Co., LTD., Tokyo, Japan) at 200 kV). Powder X-ray diffraction (XRD, Bruker optics Instruments company, Karlsruhe, Germany) was measured from 5° to 80° at room temperature on a Bruker D8 Advance diffractometer with Cu-Kα radiation and the Fourier transform-infrared spectra (FT-IR, KBr pellets, Bruker optics Instruments company, Karlsruhe, Germany) were recorded on a Nicolet 170 SXFT-IR spectrophotometer ranging from 4000 to 500 cm^−1^. X-ray photoelectron spectroscopy (XPS, Thermo Fisher Scientific, Waltham, MA, USA) was recorded on an ESCALAB 250Xi X-ray photoelectron spectrometer with an Al-Kα (*hv* = 1486.6 eV) monochromatic radiation source. The binding energy peak position of each sample was calibrated by the C 1s peak at 284.8 eV. UV-visible spectra were achieved on a Shimadzu UV–2600 UV/Vis spectrophotometer (Shimadzu Instrument Co., LTD, Kyoto-fu, Japan) using BaSO_4_ as a reference. Raman spectroscopy (Renishaw company, London, UK) was prepared using a Renishaw in Via Raman spectrometer with excitation by 325 nm Photoluminescence (PL) spectra were obtained at excitation wavelength 385 nm with TU-1900 (PuXi Company, Beijing, China).

### 2.4. Photocatalytic Hydrogen Production

Photocatalytic hydrogen producing experiments were performed using a method similar to the literature [6]. Photocatalyst (50 mg) was added to a solution of deionized water (40.0 mL) and CH_3_OH (10.0 mL, pH = 7) in a quartz vessel. The reaction system was irradiated by a 300 W Xe lamp (Perfect light PLS-SXE300, Perfectlight Technology Co., Ltd, Beijing, China), and the temperature was maintained at about 5 °C. The product H_2_ was measured by gas chromatograph (FL-9790) on line. After each photocatalytic test, photocatalysts were collected by centrifugation without reactivated before each cycle, washed with H_2_O and C_2_H_5_OH to remove impurities, then used after drying.

### 2.5. Photocatalytic Measurement

The Mott–Schottky curves were tested on an AMETEK Princeton Applied Research (Versa STAT 4, Princeton, NJ, USA) electrochemical workstation (FTO = fluorine-doped tin oxide substrate, 1 cm × 1.5 cm). The electrochemical impedance spectroscopy (EIS) and photocurrent response were recorded on an electrochemical workstation (CHI660, Chenhua, Shanghai, China) equipped with a three-electrode system with a complex/FTO as the working electrode, platinum foil as the counter electrode and Ag/AgCl as the reference electrode in 0.2 M sodium sulfate solution (Na_2_SO_4_). The working electrode complex/FTO was prepared by dropwise adding 50 μL of sample suspensions containing Nb_6_/PPy-RGO composites (3.0 mg), ethanol (1.0 mL), and nafion (20 μL) onto a FTO substrate (1 cm × 1.5 cm). 

## 3. Results and Discussion

### 3.1. Structural Characterizations

**Figure 1:**
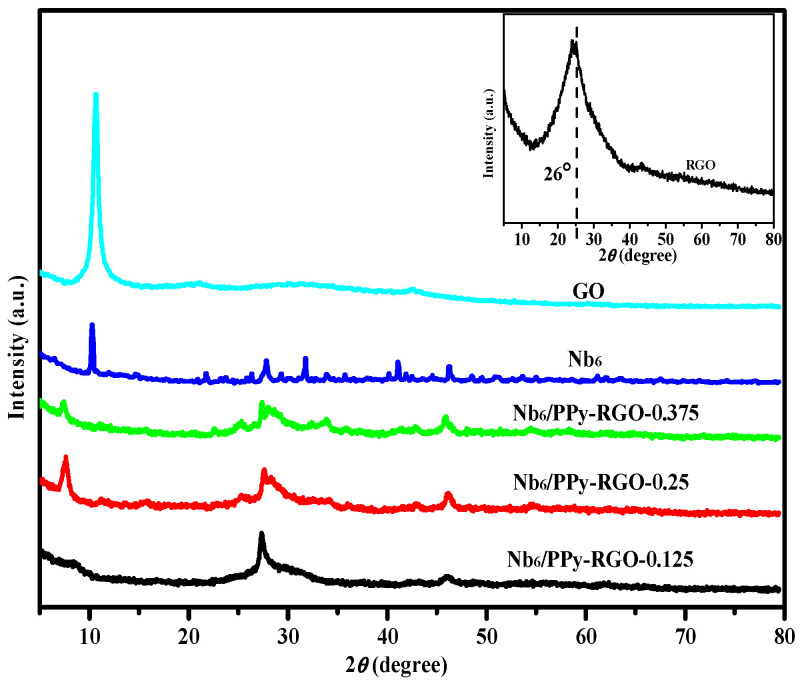
X-ray diffraction (XRD) patterns of graphene oxide (GO), RGO (insert figure), Nb_6_ and Nb_6_/PPy-RGO composites.

The XRD patterns of GO, Nb_6_, and Nb_6_/PPy-RGO composites were investigated. As shown in Figure 1 and Figure S1, pristine GO and RGO show a single peak near 12° and 26°, respectively, while pristine K_7_HNb_6_O_19_ has sharp diffraction peaks at 9.7°, 26° and 48°, respectively, which is consistent with the literature reports [46,47,48]. The broad peaks centered on 27.5° of Nb_6_/PPy-RGO composites can be attributed to the combined action of PPy and RGO, while the characteristic peaks at 46° are attributed to the superposition of Nb_6_ and PPy [49,50,51]. In Nb_6_/PPy-RGO composite materials, although it is not obvious in Nb_6_/PPy-RGO-0.325 and Nb_6_/PPy-RGO-0.25, the characteristic peaks of raw materials can still be found. No additional diffraction peaks were observed, confirming the formation of the Nb_6_/PPy-RGO composites. The crystallite sizes of the catalysts calculated from the XRD patterns were shown in Table 1.

**Figure 2:**
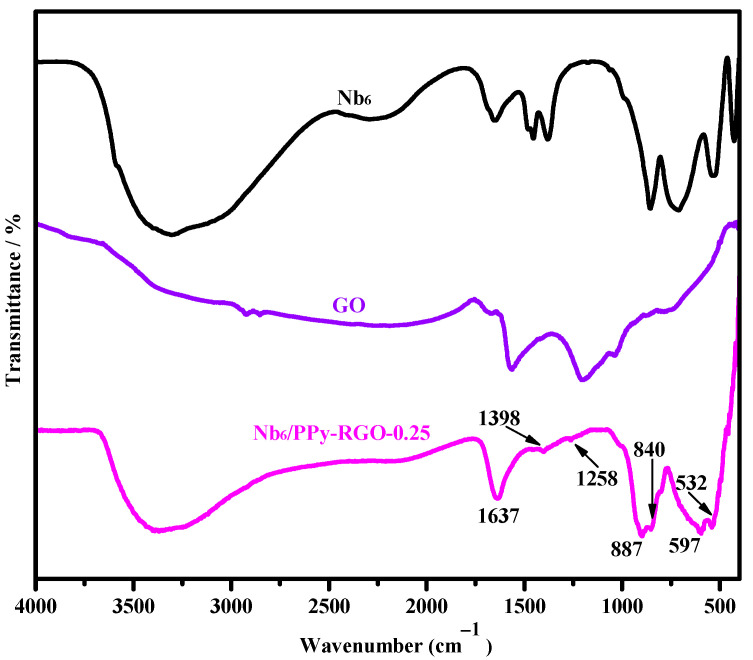
Fourier transform-infrared (FT-IR) spectra of Nb_6_, GO and Nb_6_/PPy-RGO-0.25.

The structure of Nb_6_/PPy-RGO-0.25 composite has also been evidenced by Fourier transform-infrared (FT-IR) spectroscopy (Figure 2). The peak at 1398 cm^−1^ is assigned to the C-O stretching vibration due to π–π interaction between PPy and RGO. The peak at 1637 cm^−1^ is assigned to H-O-H stretching vibration and the peak at 1258 cm^−1^ was assigned to C-H and C-N in-plane deformation vibration, further implying the combination of GO with PPy [52,53]. The characteristic peaks at 840 and 887 cm^−1^ could be attributed to the terminal Nb-O stretching vibration, and the peaks appearing at 532 and 597 cm^−1^ correspond to Nb-O-Nb bonds [54]. The result shows that Nb_6_ was successfully inserted into the final composite. 

**Figure 3:**
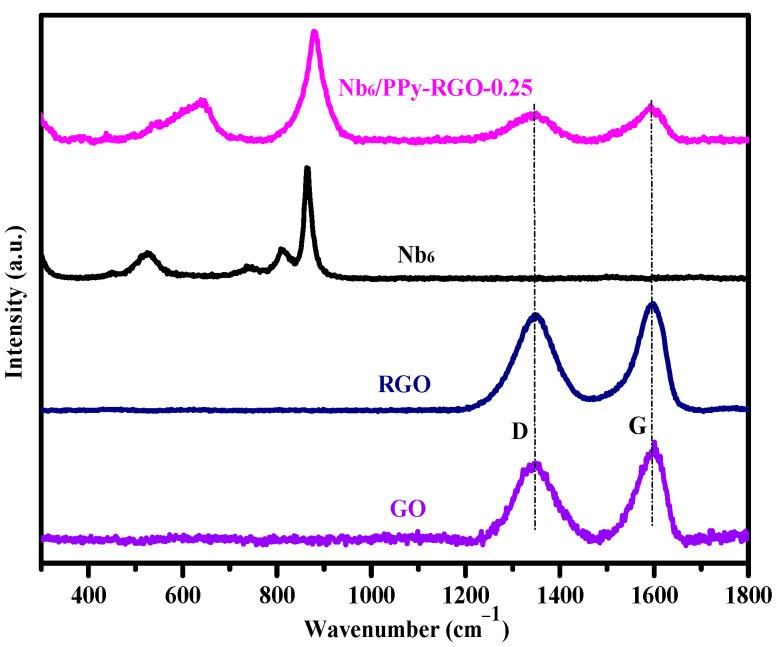
Raman spectra of Nb_6_, GO, RGO and Nb_6_/PPy-RGO-0.25.

From the Raman spectra shown in Figure 3, the higher wavenumber bands at 1334 and 1584 cm^−1^ are ascribed to D band and G band of GO. It is worth noting that the peak intensity ratio (*I*_D_/*I*_G_) is a popular method to evaluate the disorder and reduction of graphene materials. The *I*_D_/*I*_G_ ratio of the pure GO is about 0.76, while that of the Nb_6_/PPy-RGO-0.25 composite is about 0.99. The numeric addition indicated that the oxygen-containing functional groups were partially reduced, meaning the transition of GO to RGO in the process of solvothermal reaction [54]. Compared with the characteristic peaks of Nb_6_ (541, 827 and 875 cm^−1^) [55,56], the redshift could be found for Nb_6_/PPy-RGO-0.25 (878 and 620 cm^−1^), which may be assigned to the strong interaction among the Nb_6_, PPy, and RGO of the composites.

**Figure 4:**
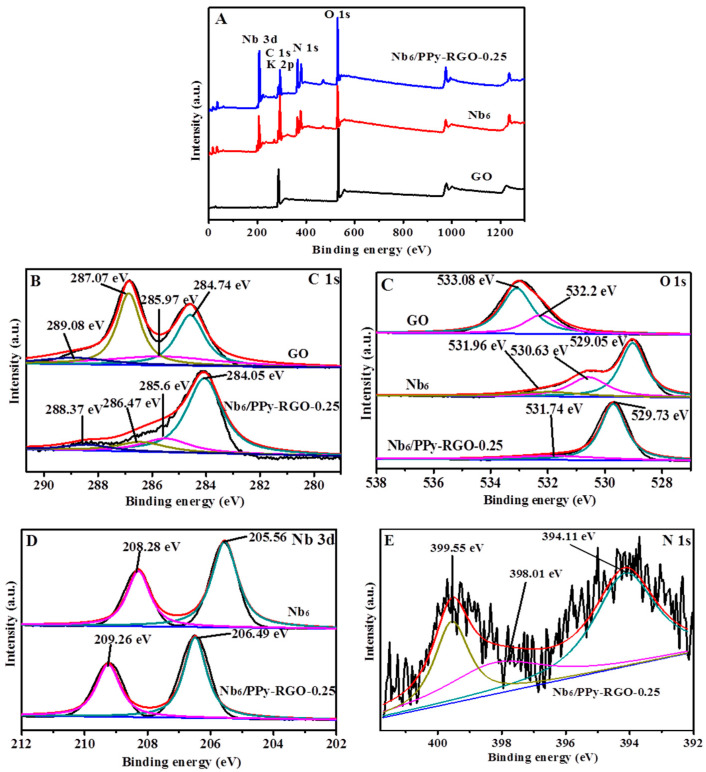
X-ray photoelectron spectroscopy (XPS) of GO, Nb_6_, and Nb_6_/PPy-RGO-0.25, (**A**) survey; (**B**) C 1s; (**C**) O 1s; (**D**) Nb 3d; (**E**) N 1s.

In order to further identify the structure of composites, the XPS spectra were tested for Nb_6_/PPy-RGO-0.25 composites and GO, as well as Nb_6_ (Figure 4). As shown in Figure 4A, the full spectrum clearly shows the presence of C, O, N, K, and Nb elements in the composite sample, which is consistent with the chemical composition of the photocatalyst. As shown in Figure 4B, the binding energies of C 1s, including 284.05, 285.60, 286.47 and 288.37 eV, could be attributed to carbon of the non-oxygenated ring, C-OH, C-O and C=O in Nb_6_/PPy-RGO-0.25, respectively, which are lower and weaker than that of pure GO (284.74, 285.97, 287.07 and 289.08 eV). These results demonstrate that the GO has been successfully reduced in composites, which is in agreement with Raman spectra [57,58,59,60]. For pure GO, the XPS of O 1s contains two peaks at 532.20 and 533.08 eV assigned to C=O/C-O and O-C=O, respectively. The peaks at 531.96, 529.05, and 530.63 eV in the pure Nb_6_ sample belong to Nb-O-Nb, Nb-O-H, and crystal water, respectively. There are two main peaks at 529.73 and 531.74 eV in Nb_6_/PPy-RGO-0.25, which can be attributed to binding energies of O 1s in Nb-O-Nb and Nb-O-H (Figure 4C [61]. Figure 4D illustrates a comprehensive Nb 3d XPS analysis of the Nb_6_/PPy-RGO-0.25 and Nb_6_. The binding energies of 206.49 and 209.26 eV could be assigned to Nb 3d_5/2_ and Nb 3d_3/2_ of Nb^5+^ (highest oxidation state) in Nb_6_/PPy-RGO-0.25. Compared with Nb_6_, the peaks of Nb 3d shifted to higher binding energy by ~1 eV, which may be caused by electron transfer from Nb_6_ to RGO in composites [54]. As shown in Figure 4E, the N 1s peaks centered at 398.01 and 399.55 eV are vested to the pyridinic and pyrrolic of N atom in polymer. In addition, there is a weak peak at the center of 394.11 eV, corresponding to the Nb-N bond, indicating the formation of chemical bonds between PPy and Nb_6_ [62].

### 3.2. Nanosphere Morphologies

**Figure 5:**
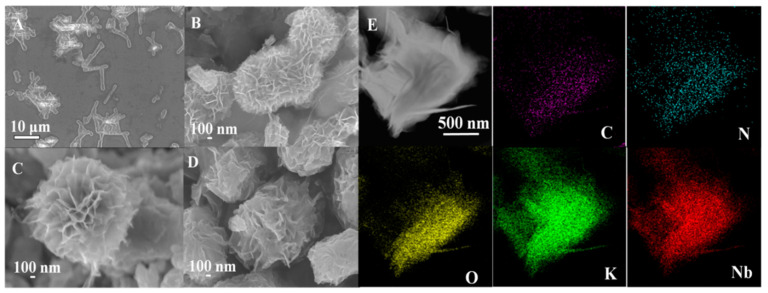
Scanning electron microscopy (SEM) images for: (**A**) Nb_6_; (**B**) Nb_6_/PPy-RGO-0.125; (**C**) Nb_6_/PPy-RGO-0.25; (**D**) Nb_6_/PPy-RGO-0.375. (**E**) Transmission electron microscopy (TEM) images and the element maps of Nb_6_/PPy-RGO-0.25.

As shown in Figure 5, the results show that the morphology of Nb_6_ is rodlike. However, when fabricated with PPy and RGO, Nb_6_/PPy-RGO-X (X = 0.125, 0.25, 0.375) display the flower-like methodology. To deeply understand the formation of flower-like methodology, the controlled experiments including solvothermal reaction temperatures and the amount of Py have been respectively conducted using Nb_6_/PPy-RGO-0.25 as a representative. As shown in Figures S2 and S3, the samples probably agglomerated when changing the conditions of 160 °C or Py (130 μL). However, the lower amount of Py, with 90 μL, or higher temperature of 190 °C may reduce the surface area and could not form morphology of flowers for Nb_6_/PPy-RGO-0.25. These results demonstrate that the flower-like morphology for Nb_6_/PPy-RGO-0.25 largely depends on the temperature and the amounts of Py in the reaction. The corresponding EDS elemental mapping of Nb_6_/PPy-RGO-0.25 indicated the elements of C, N, O, K and Nb were uniformly distributed throughout the whole composite (Figure 5E).

### 3.3. Photochemistry and Electrochemistry

**Figure 6:**
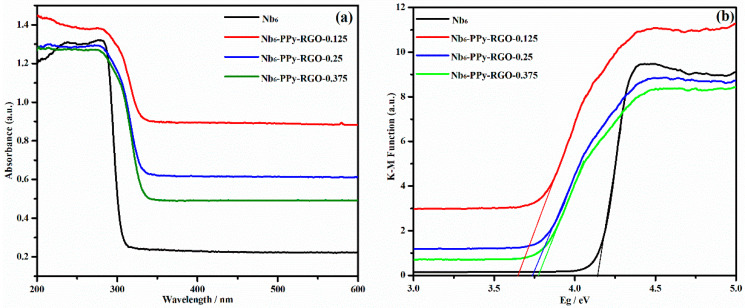
(**a**) UV-vis diffuses reflectance spectra of Nb_6_ and Nb_6_/PPy-RGO composites; (**b**) Kubelka–Munk function-calculated band gap of Nb_6_ and Nb_6_/PPy-RGO composites.

The UV-Vis diffuse reflectance spectra of Nb_6_/PPy-RGO composites were recorded at room temperature to discuss their optical properties, together with Nb_6_ for comparison. As shown in Figure 6, Nb_6_/PPy-RGO composites exhibit broad absorptions in the range of 200–340 nm. Compared with the absorption of Nb_6_ (200–315 nm), the light absorption of Nb_6_/PPy-RGO samples was obviously widened, which is related to the introduction of PPy and RGO. Due to the zero band gap for RGO, with the increasing amounts of Nb_6_, the absorbance intensity for Nb_6_/PPy-RGO composites become weaker (Figure 6a). Simultaneously, the color becomes lighter and the ability to capture light is decreased (Figure S4), hinting that the enhanced absorption is mainly attributed to the introduction of RGO. The band-gap values of Nb_6_ and Nb_6_/PPy-RGO composites (from 0.125 to 0.375) obtained by the Kubelka–Munk function via the UV-Vis diffuse reflectance are 4.14, 3.66, 3.71, and 3.76 eV, respectively, indicating the semiconductor characters of Nb_6_/PPy-RGO composites (Figure 6b). Compared to the band gap value of Nb_6_, those of Nb_6_/PPy-RGO composites are obviously narrowed through the incorporation of RGO and PPy.

Furthermore, the potentials of conduction band (CB) of Nb_6_/PPy-RGO composites could be obtained from electrochemical Mott–Schottky plots (Figure S5). The plots of C^−2^ vs. potential exhibited positive slopes, indicating that we should consider the composites as typical n-type semiconductors [57]. The flat band position of Nb_6_/PPy-RGO composites determined from the intersection of the Mott–Schottky plot and X axis are approximately −1.01 V (Nb_6_/PPy-RGO-0.125), −1.34 V (Nb_6_/PPy-RGO-0.25) and −1.23 V (Nb_6_/PPy-RGO-0.375) (vs. NHE, NHE = Normal hydrogen electrode). These results imply that the potentials of CB of Nb_6_/PPy-RGO composites are more negative than the redox potential of H^+^/H_2_ (−0.41 V vs. NHE, pH = 7) [63], indicating the title composites should be used as photocatalysts for hydrogen evolution via water splitting.

**Figure 7:**
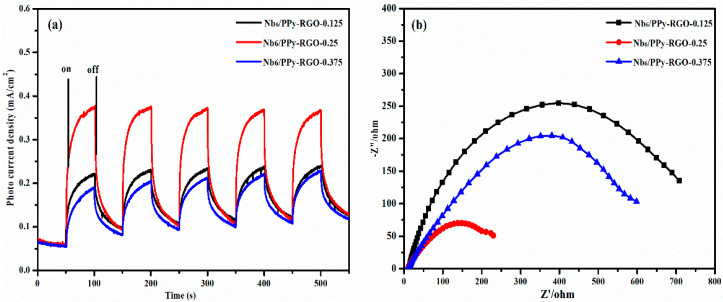
(**a**) Transient photocurrent response and (**b**) electrochemical impedance spectroscopy (EIS) plots of Nb_6_/PPy-RGO-0.125, Nb_6_/PPy-RGO-0.25 and Nb_6_/PPy-RGO-0.375.

As shown in Figure S6, compared with other composites, the Nb_6_/PPy-RGO-0.25 composite displays the weakest PL intensity at 378 nm, and the amount of pyrrole and the solvothermal temperature had little effect on the separation of photo-excited charge and holes, indicative of the lowest combination of electron-hole pairs. Because K_7_HNb_6_O_19_ has good water solubility, electrochemical measurement cannot be performed under the same conditions. As shown in Figure 7a, the highest photocurrent response of Nb_6_/PPy-RGO-0.25 shows better separation ability and lower recombination of photogenerated electron-hole pairs [42]. As depicted in Figure 7b, the smallest radius of Nb_6_/PPy-RGO-0.25 indicates lower charge transfer resistance. This result confirms that more photo-generated charge carriers can be separated by constructing Nb_6_/PPy-RGO-0.25, which is consistent with the result of PL analysis and photocurrent response.

### 3.4. Photocatalytic Hydrogen Evolution

**Figure 8:**
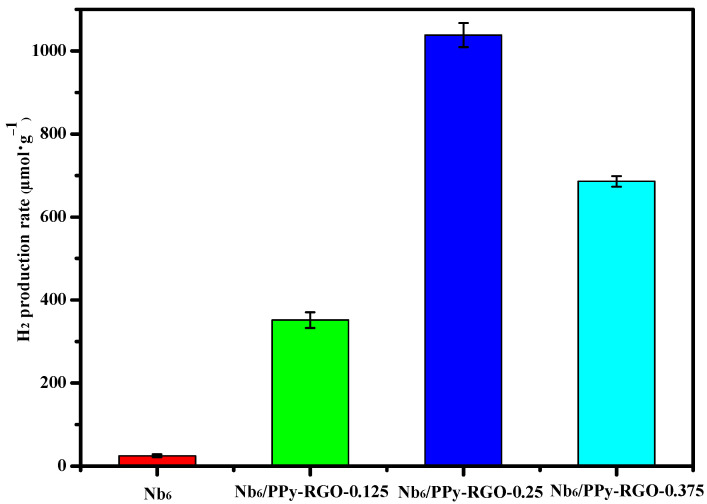
Hydrogen evolution of Nb_6_ and Nb_6_/PPy-RGO composites in aqueous solution with MeOH 20%.

Photocatalytic activity of Nb_6_/PPy-RGO composites for hydrogen evolution via water splitting was evaluated in aqueous solution with methanol (CH_3_OH) as a sacrificial electron donor. As shown in Figure 8, the H_2_ production for Nb_6_/PPy-RGO composites was 351.5 (Nb_6_/PPy-RGO-0.125), 1038 (Nb_6_/PPy-RGO-0.25) and 690.5 μmol g^−1^ (Nb_6_/PPy-RGO-0.375) in 5 h, respectively. Under the same conditions, the H_2_ production was 25 μmol g^−1^ for the Nb_6_ catalyst. Apparently, the composites exhibit higher photocatalytic activity by introduction of PPy and RGO. Compared with the other two composites, Nb_6_/PPy-RGO-0.125 and Nb_6_/PPy-RGO-0.375, Nb_6_/PPy-RGO-0.25 exhibits better photocatalytic efficiency and the turnover frequency (TOF) value reaches 830.4 × 10^−3^ h^−1^ (Table 2). These results agree well with EIS and photo-current measurements.

Turnover frequency (TOF) was based on Nb_6_, which has been widely used to evaluate the property of catalysts, especially for POM-based catalysts. The details are as follows:

(1) $$\mathrm{TOF}\;=\;\frac{n_{\left(H_{2}\right)}/h}{n_{\left(Nb_{6}\right)}}$$

Taking Nb_6_/PPy-RGO-0.25 as an example:

(2) $${\mathrm{TOF}}_{{(\mathrm{Nb}}_{6}/\mathrm{PPy}-\mathrm{RGO}-0.25)}=\frac{n_{\left(H_{2}\right)}/h}{n_{\left(Nb_{6}\right)}}=\frac{1038\times 10^{-6}\mathrm{mol}/5h}{0.25\times 10^{-3}\mathrm{mol}}=830.4\times 10^{-3}h^{-1}$$

From the above table, the sample Nb_6_/PPy-RGO-0.25 showed the better photocatalytic activity. 

The photocatalytic system has also been optimized by changing the amount or species of sacrificial electron donors by using the optimal as Nb_6_/PPy-RGO-0.25 photocatalyst. As shown in Figure S7a, when the concentration of CH_3_OH increased from 0 to 20% (*v*:*v*), the hydrogen evolution increased from 0 to 1038 μmol g^−1^, but decreased to 274 and 94 μmol g^−1^ with higher concentrations of 25% (*v*:*v*) and 30% (*v*:*v*), respectively. These results imply that the amount of CH_3_OH played an important role in photocatalytic hydrogen evolution. The more sacrificial electron donors may react with holes of photocatalyst to release more photo-excited electrons to produce more hydrogen. However, with an excess of CH_3_OH, it should also decrease the concentrations of substrate, leading to lower photocatalytic efficiency. Furthermore, the species of sacrificial electron donor was optimized by controlling the ratio of sacrificial agent/H_2_O (*v*:*v* = 10:40) (Figure S7b). The title photocatalyst exhibited the optimal H_2_ production of 1038 μmol g^−1^ with CH_3_OH as the sacrificial agent, but varied in the range of 249.5–834 μmol g^−1^ with other sacrificial agents. As shown in Figure S8, the best sample, Nb_6_/PPy-RGO-0.25, exhibits the highest photocatalytic H_2_ production, indicating the optimal reaction conditions with pyrrole dosage of 110 μL and reaction temperature of 180 °C. 

**Figure 9:**
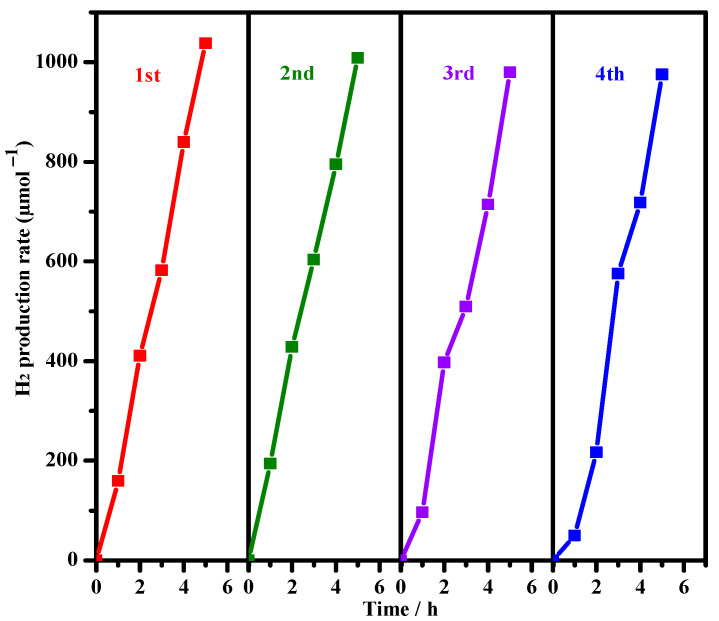
Changes in photocatalytic performance of Nb_6_/PPy-RGO-0.25 during the recycled uses.

In order to prove the reusability of Nb_6_/PPy-RGO-0.25, the recycling experiment was carried out (Figure 9). There was no noticeable deactivation observed for the catalyst and the yields of H_2_ evolution for the four recycling experiments all remained at 1000 μmol g^−1^ in 5 h. In addition, the XRD pattern (Figure S9), Raman spectra (Figure S10) and SEM image (Figure S11) of the recovered catalysts were alike to those of the as-prepared sample, further demonstrating the recyclability and stability of Nb_6_/PPy-RGO-0.25 in the photocatalysis reaction.

### 3.5. Proposed Photocatalytic Mechanism

**Figure 10:**
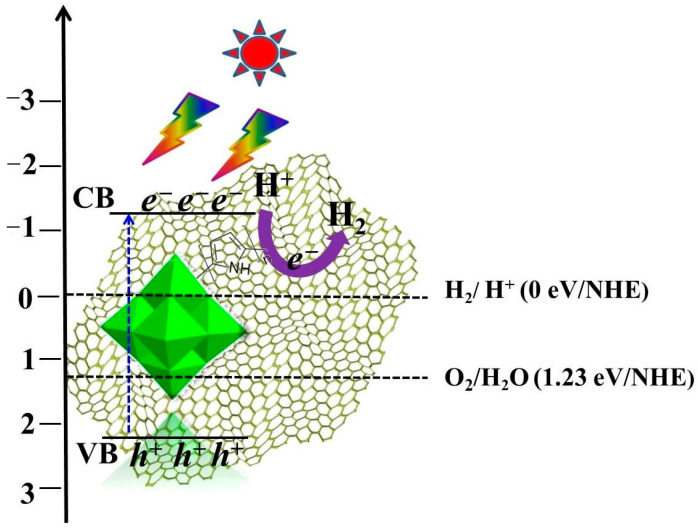
The proposed photocatalytic hydrogen production mechanism of Nb_6_/PPy-RGO-0.25 (NHE = normal hydrogen electrode).

According to the Mott–Schottky plot shown in Figure S5, the conduction band minimum (CBM) of the composites could be estimated to explain the enhanced photocatalytic activity mechanism, which is about −1.34 eV for the sample Nb_6_/PPy-RGO-0.25 possessing the best H_2_ evolution performance. Meanwhile, valence band maximum (VBM) potential of Nb_6_/PPy-RGO-0.25 can be determined based on the formula *E*_VBM_ = *E*_CBM_ + *E*_g_, which is about 2.37 eV [64]. As displayed in Figure 10, the pure Nb_6_ exhibits negligible photocatalytic activity, which may be due to the fast recombination of electrons and holes. Higher photocatalytic efficiency can be achieved for Nb_6_/PPy-RGO composites by the modification with PPy and RGO. It has been reported that conductive polymers can be used as photocatalysts for H_2_ production from water splitting [65]. However, in such Nb_6_/PPy-RGO-0.25 composites, pure PPy and RGO cannot exhibit photocatalytic activity due to their combination of electrons and holes. On the other hand, they can prolong the lifetime of charge carriers to promote the separation efficiency of electron-hole pairs in Nb_6_/PPy-RGO-0.25 composites due to their excellent π conjugated chain structure and electrical conductivity. In addition, RGO can also serve as active sites to produce H_2_, avoiding the use of noble metals, while PPy acted as conductive polymers, promoting the transfer of the electrons [52,53,66,67,68,69]. In particular, the π–π interaction between RGO and PPy further strengthens the electrical conductivity. Based on the above results, a probable photocatalytic mechanism for composites could be proposed as follows: under light irradiation, the electrons in the valence band (VB) of Nb_6_/PPy-RGO-0.25 can be excited to the CB, causing holes in the VB. On one hand, the holes in the VB could be quenched by accepting electrons from CH_3_OH. On the other hand, the photoelectron in the CB of Nb_6_/PPy-RGO-0.25 could be transferred to RGO through the bridge of PPy. Acting as photoactive sites, the RGO accepting electrons would react with H_2_O to produce H_2_. 

## 4. Conclusions

In summary, a series of unprecedented polyoxoniobate-graphene nanocomposites, Nb_6_/PPy-RGO, have been successfully synthesized through a simple one-step solvothermal method. This is the first example of polyoxoniobate-graphene nanocomposites for photocatalytic H_2_ production. This work presents a new idea for the design and synthesis of high-performance polyoxometalates-graphene photocatalysts in photocatalytic H_2_ production. Nb_6_/PPy-RGO was optimized by adjusting the amount of Nb_6_, volume of pyrrole and temperature, displaying superb photocatalytic H_2_ production of 1038 μmol g^−1^ in 5 h without a co-catalyst, almost 43 times more than pure Nb_6_, in which the collaborations among Nb_6_, PPy and RGO play vital roles in such a photocatalytic system. In further work, we will focus on studying more fascinating polyoxoniobate-graphene nanocomposites with new interesting properties.

## Figures and Tables

**Table 1:** The crystallite sizes of Nb_6_ and Nb_6_/PPy-RGO composites (Nb_6_ = K_7_HNb_6_O_19_, RGO = reduced graphene oxide, PPy = polypyrrole).

Samples	Nb_6_	Nb_6_/PPy-RGO-0.125	Nb_6_/PPy-RGO-0.25	Nb_6_/PPy-RGO-0.375
Crystalite sizes (nm)	26	6	12	14

**Table 2:** The turnover frequency (TOF) values of Nb_6_ and Nb_6_/PPy-RGO composites.

Samples	Nb_6_	Nb_6_/PPy-RGO-0.125	Nb_6_/PPy-RGO-0.25	Nb_6_/PPy-RGO-0.375
TOF (h^−1^)	137 × 10^−3^	562.6 × 10^−3^	830.4 × 10^−3^	368 × 10^−3^

## Supplementary Materials

The following are available online at https://www.mdpi.com/2079-4991/10/12/2449/s1, Figure S1: X-ray diffraction (XRD) patterns for series of concentration of pyrrole and temperature and the corresponding starting materials. Figure S2: SEM images for (**a**) Nb_6_-RGO; (**b**) Nb_6_/PPy-RGO-90 μL; (**c**) Nb_6_/PPy-RGO-110 μL; (**d**) Nb_6_/PPy-RGO-130 μL (SEM = scanning electron microscopy, Nb_6_ = K_7_HNb_6_O_19_, RGO = reduced graphene oxide, PPy = polypyrrole). Figure S3: SEM images for (**a**) Nb_6_/PPy-RGO-160 °C; (**b**) Nb_6_/PPy-RGO-180°C; (**c**) Nb_6_/PPy-RGO-190 °C. Figure S4: The color change of samples. Figure S5: Mott-Schottky plots of (**a**) Nb_6_/PPy-RGO-0.125; (**b**) Nb_6_/PPy-RGO-0.25; (**c**) Nb_6_/PPy-RGO-0.375, in 0.2 M Na_2_SO_4_ aqueous solution with pH = 7. Figure S6: Photoluminescence (PL) spectra recorded at room temperature in the range of 325–450 nm with an excitation wavelength of 378 nm for adjust (**a**) molar ratio of Nb_6_; (**b**) volume of pyrrole; (**c**) temperature. Figure S7: (**a**) Rate of H_2_ evolution as a function of CH_3_OH concentration, (**b**) Rate of H_2_ evolution as a function of type of sacrificial agents. Figure S8: Photocatalytic hydrogen production in concentration of pyrrole (**a**) and temperature (**b**) in aqueous solution with MeOH 20%. Figure S9: XRD patterns of Nb_6_/PPy-RGO-0.25 before and after photocatalytic H_2_ evolution reaction. Figure S10: Raman spectra of Nb_6_/PPy-RGO-0.25 before and after photocatalytic H_2_ evolution reaction. Figure S11: SEM images for (**a**) Nb_6_/PPy-RGO-0.25 and (**b**) that after the recycle of photocatalytic hydrogen evaluation.
