# Supplementary material for: Enhanced Photocatalytic Hydrogen Production of the Polyoxoniobate Modified with RGO and PPy
Source: Nanomaterials (Basel). 2020 Dec 7;10(12):2449. doi: 10.3390/nano10122449 (PMC7762403; doi:10.3390/nano10122449)
Supplement: Supplementary file 1 [file nanomaterials-10-02449-s001.pdf]

nanomaterials

Supplementary Materials

Enhanced Photocatalytic Hydrogen Production of the Polyoxoniobate Modified with RGO and PPy

Shiliang Heng [1], Lei Li [1,2], Weiwei Li [1], Haiyan Li [1], Jingyu Pang [1], Mengzhen Zhang [1], Yan Bai [1,*] and Dongbin Dang [1,*]

[1] Henan Key Laboratory of Polyoxometalate Chemistry, College of Chemistry and Chemical Engineering, Henan University, Kaifeng 475004, China; 104753170767@vip.henu.edu.cn (S.H.); leili1202@126.com (L.L.); 104753160776@vip.henu.edu.cn (W.L.); lihaiyan@henu.edu.cn (H.L.); pjy@henu.edu.cn (J.P.); 104754181031@vip.henu.edu.cn (M.Z.)

[2] College of Chemistry and Chemical Engineering, Anyang Normal University, Anyang 455002, China

* Correspondence: baiyan@henu.edu.cn (Y.B.); dangdb@henu.edu.cn (D.D.)

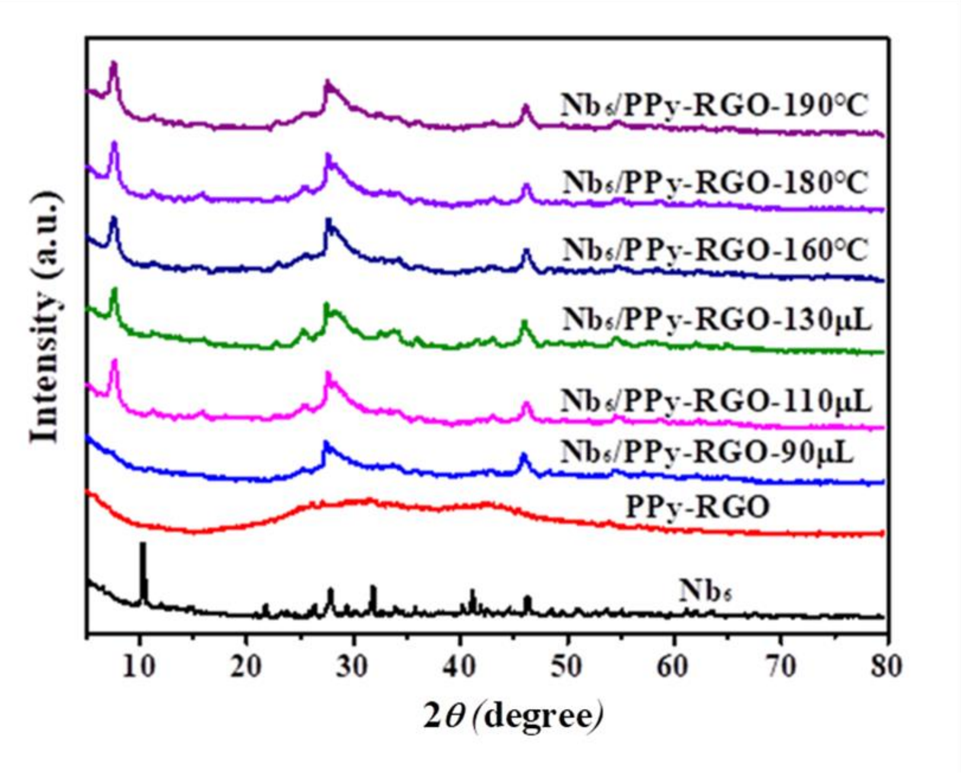

Figure S1. X-ray diffraction (XRD) patterns for series of concentration of pyrrole and temperature and the corresponding starting materials.

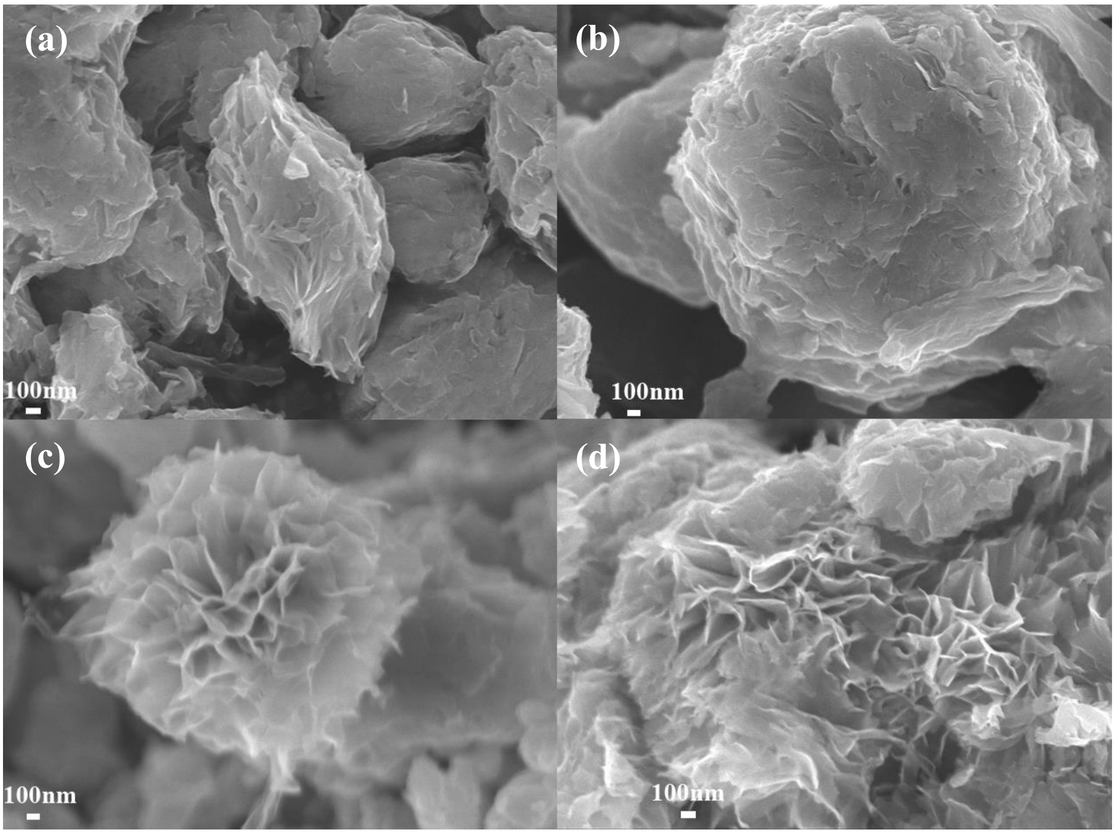

Figure S2. SEM images for: (**a**) Nb_6-RGO; (**b**) Nb_6/PPy-RGO-90 μL; (**c**) Nb_6/PPy-RGO-110 μL; (**d**) Nb_6/PPy-RGO-130 μL. (SEM = scanning electron microscopy, Nb_6 = $K_7HNb_6O_{19}$, RGO = reduced graphene oxide, PPy = polypyrrole).

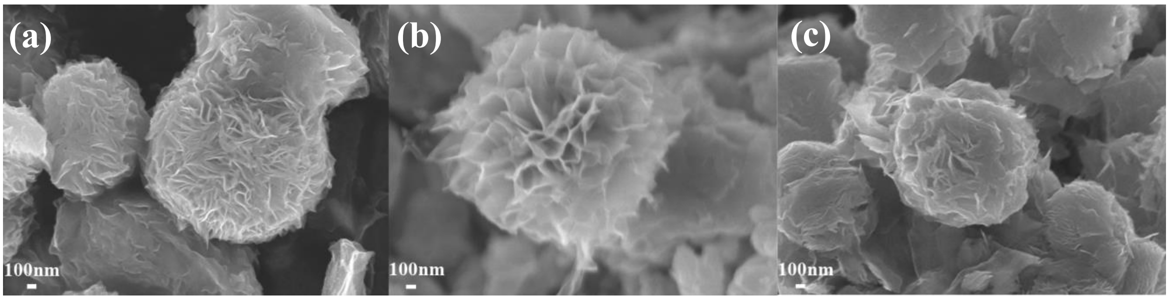

Figure S3. SEM images for: (**a**) Nb_6/PPy-RGO-160 °C; (**b**) Nb_6/PPy-RGO-180 °C; (**c**) Nb_6/PPy-RGO-190 °C.

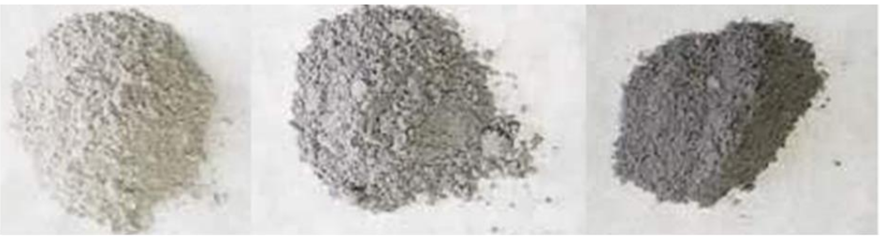

Nb6/PPy-RGO-0.375   Nb6/PPy-RGO-0.25   Nb6/PPy-RGO-0.125

Figure S4. The color change of samples.

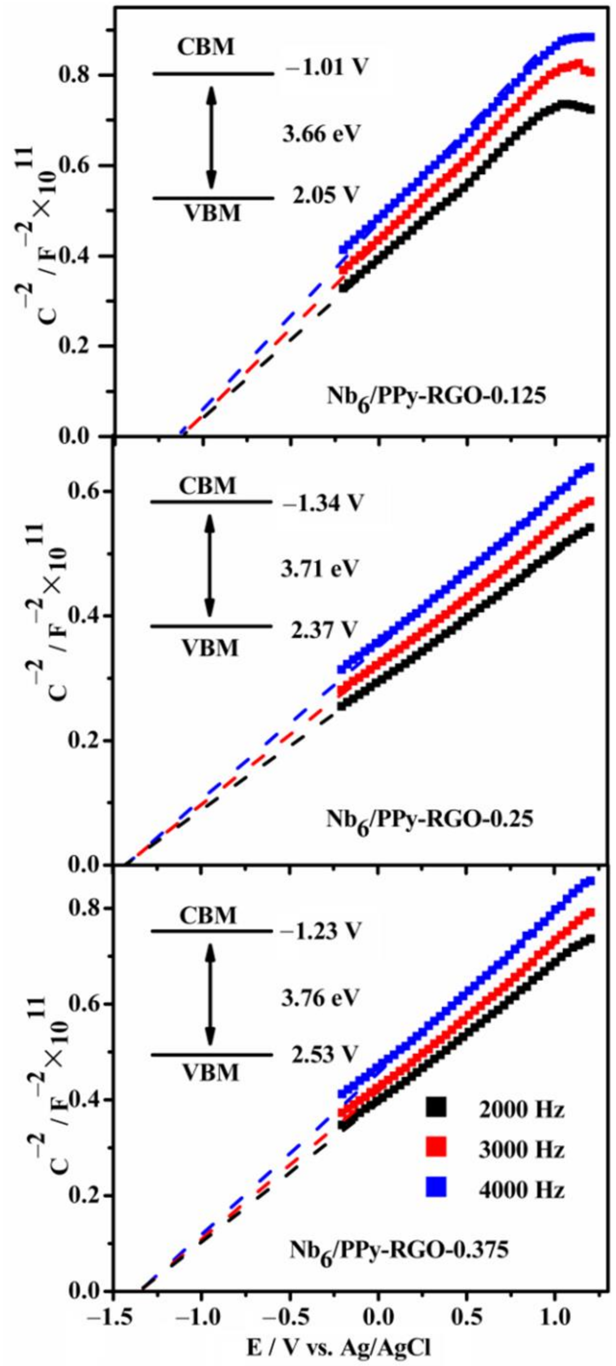

Figure S5. Mott-Schottky plots of: (**a**) Nb_6/PPy-RGO-0.125; (**b**) Nb_6/PPy-RGO-0.25; (**c**) Nb_6/PPy-RGO-0.375, in 0.2 M Na_2SO_4 aqueous solution with pH = 7.

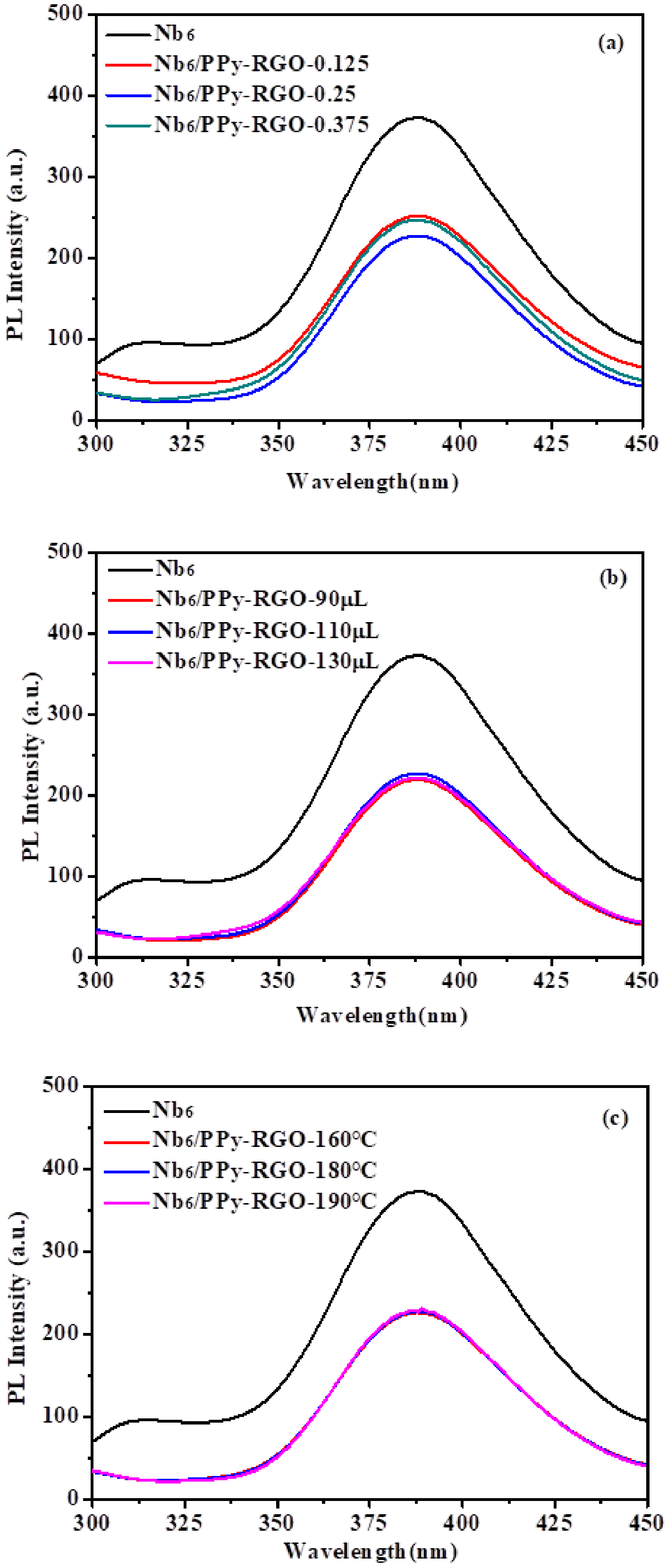

Figure S6. Photoluminescence (PL) spectra recorded at room temperature in the range of 325–450 nm with an excitation wavelength of 378 nm for adjust (**a**) molar ratio of Nb_6; (**b**) volume of pyrrole; (**c**) temperature.

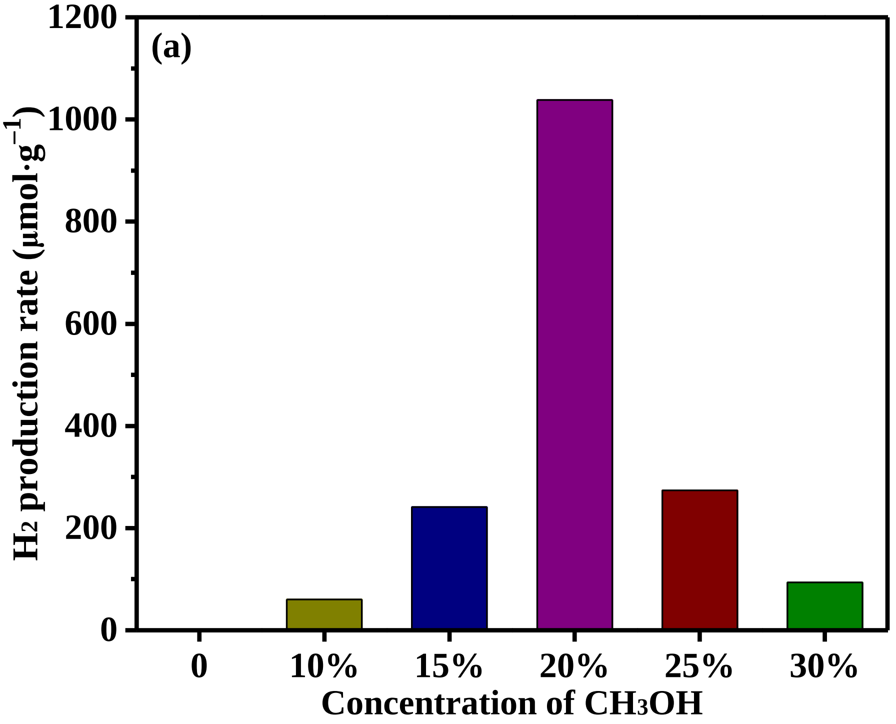

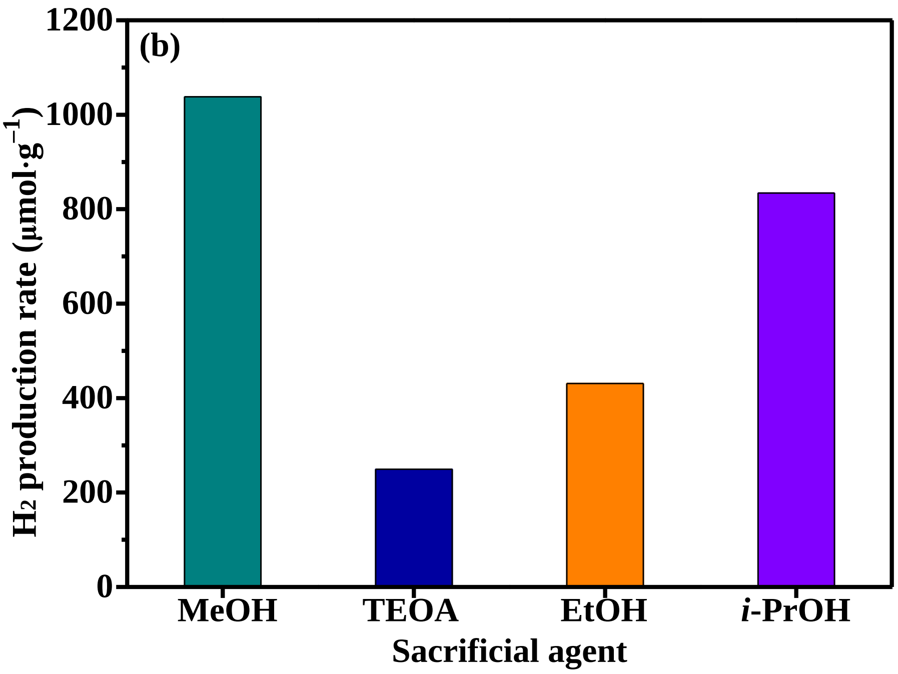

Figure S7. (**a**) Rate of H_2 evolution as a function of CH_3OH concentration; (**b**) Rate of H_2 evolution as a function of type of sacrificial agents.

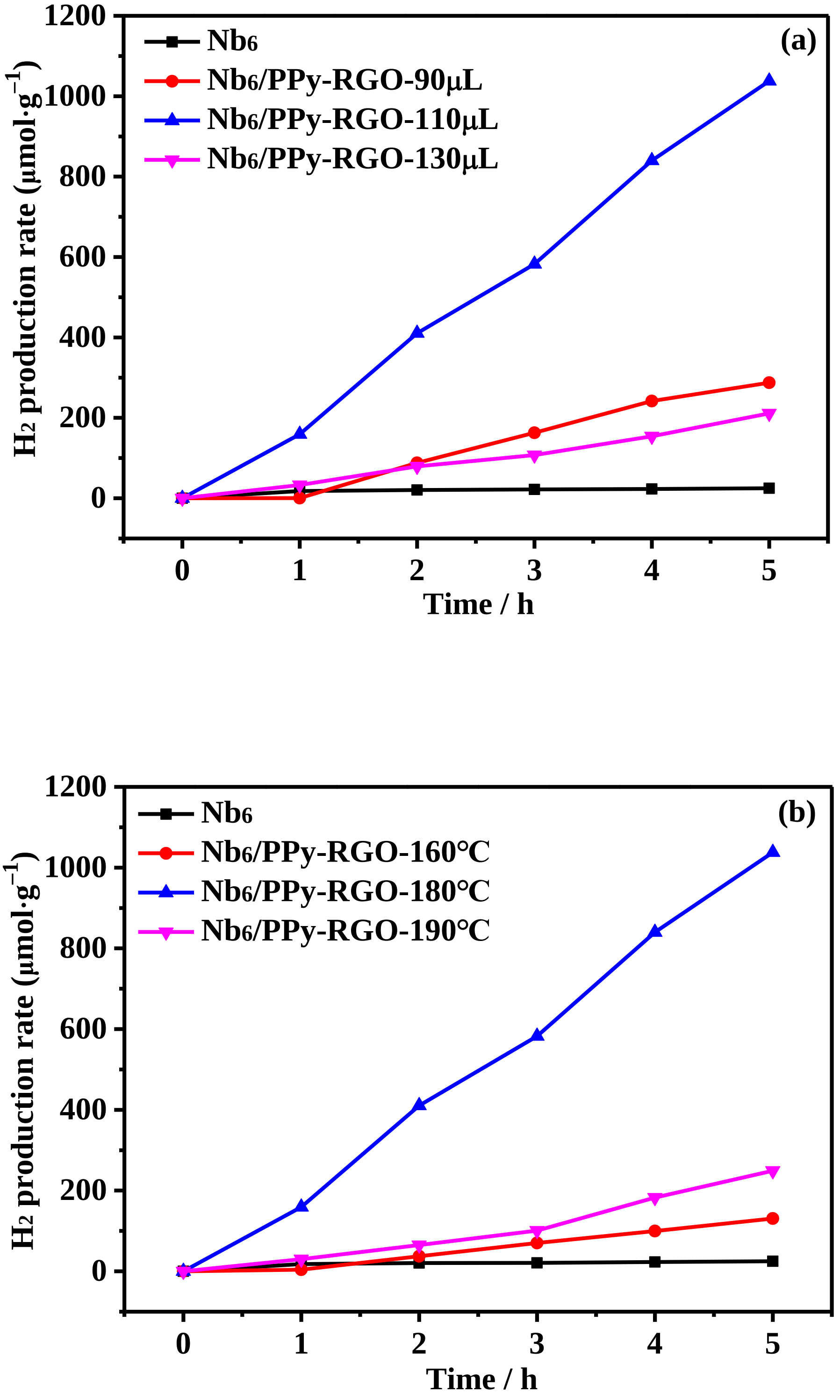

Figure S8. (**a**) Photocatalytic hydrogen production in concentration of pyrrole and (**b**) temperature in aqueous solution with MeOH 20%.

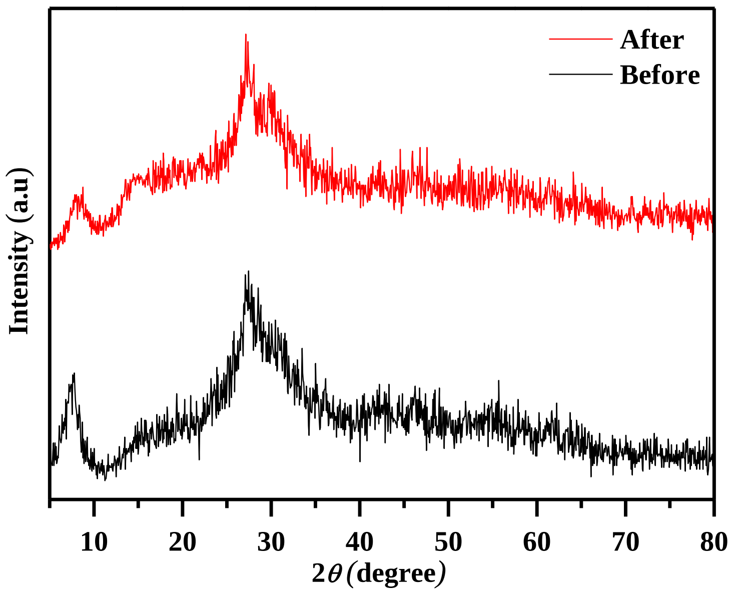

Figure S9. XRD patterns of Nb_6/PPy-RGO-0.25 before and after of photocatalytic H_2 evolution reaction.

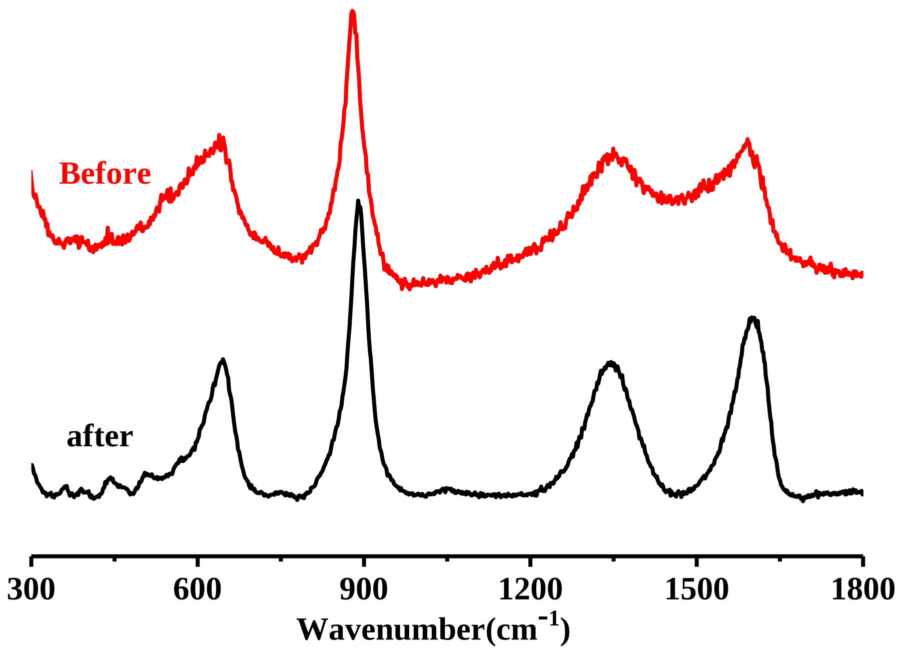

Figure S10. Raman spectra of Nb_6/PPy-RGO-0.25 before and after of photocatalytic H_2 evolution reaction.

.

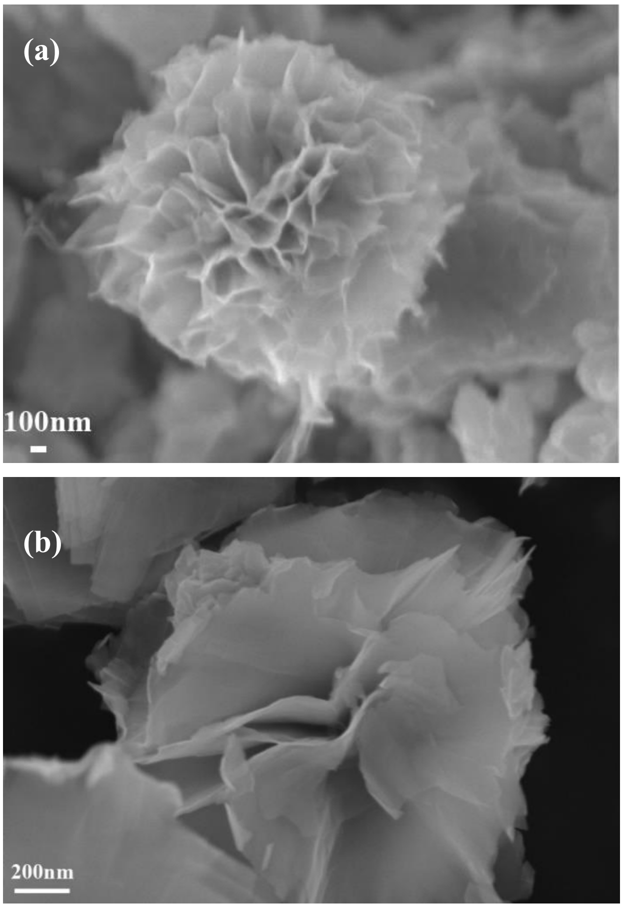

Figure S11. SEM images for (**a**) Nb_6/PPy-RGO-0.25 and (**b**) that after the recycle of photocatalytic hydrogen evaluation.
